# Comprehensive evaluation of the risk of lactational mastitis in Chinese women: combined logistic regression analysis with receiver operating characteristic curve

**DOI:** 10.1042/BSR20190919

**Published:** 2020-03-20

**Authors:** Yongshuo Yin, Zhiyong Yu, Min Zhao, Yuemei Wang, Xiao Guan

**Affiliations:** 1 Department of Oncology, Shandong Cancer Hospital affiliated to Shandong University, Shandong Academy of Medical Sciences, Jinan City, Shandong Province, China; 2 Department of Nutrition and Food Hygiene, School of Public Health, Shandong University, Jinan City, Shandong Province, China; 3 Department of Ultrasonography, Jinan Maternity and Child Care Hospital, Jinan City, Shandong Province, China; 4 Department of Health Management Center, Qilu Hospital of Shandong University, Jinan City, Shandong Province, China

**Keywords:** Breastfeeding, Logistic Regression, Mastitis, Risk factors, ROC curve

## Abstract

**Objective:** To identify the potential risk factors for acute mastitis during lactation comprehensively. Subsequently, to evaluate logistic regression model in predicting the risk of lactational mastitis in Chinese women by applying receiver operating characteristic (ROC) curve.

**Methods:** A case–control study among Chinese women enrolled 652 patients with mastitis and 581 healthy women with breastfeeding experience as control. The retrospective information was obtained by questionnaires that included medical history of pregnancy, delivery, puerperium and breastfeeding behaviors. Univariate analysis and multivariate logistic regression model were performed to investigate the relationship between these factors and the occurrence of lactational mastitis. Using ROC curve to evaluate the prognostic value of these selected indicators in the risk of acute mastitis.

**Results:** The multivariate logistic regression analysis showed that the primiparity (*P* < 0.001), mastitis in previous breastfeeding (*P* < 0.001), nipple’s heteroplasia (*P* < 0.001), cracked nipple (*P* < 0.001), breast trauma by external force (*P* = 0.002), lateral position (*P* = 0.007), breast pump (*P* = 0.039), nipple sucking (*P* = 0.007), sleep with sucking (*P* = 0.007), and tongue-tie (*P* = 0.013) were risk variables independently and significantly related with mastitis. While vaginal delivery (*P* = 0.015), clean nipple before breastfeeding (*P* = 0.015), first contact with child within 1 h (*P* = 0.027) were protective factors. The ROC analysis demonstrated that the area under the curve of model 2 was 0.8122 (95%CI = 0.7885–0.8360), which stated that the model presented a high sensitivity and specificity.

**Conclusion:** By means of collecting and summarizing the risk factors associated with the occurrence of breast mastitis in Chinese women, we established risk discriminant model to identify and warn the individuals susceptible to acute mastitis early, which will allow practitioners to provide appropriate management advice and effective individual care.

## Introduction

Breastfeeding is a natural infant feeding method highly recommended by the World Health Organization (WHO) [1] and the United Nations International Children’s Emergency Fund (UNICEF) to ensure the healthy growth of infants and children throughout the world [2]. Furthermore, some countries have established the breastfed infant as the norm against which to assess compliance with children’s right to achieve their full genetic growth potential [3]. At present, breastfeeding is now recommended for at least 2 years [1]. The main reasons leading to failure of breastfeeding are acute mastitis and breast abscess [4]. Approximately 3–33% of breastfeeding women suffer from mastitis, particularly in the 6 months after childbirth [5,6]. The reason is that there are no interventions that have been consistently proven effective for preventing mastitis. Encouraging emptying milk from the breast, avoiding nipples damage and taking antibiotics may reduce the risk of developing mastitis. Even so, about 3–11% of acute mastitis are still prone to develop to breast abscess in the case of rapid progression and improper treatment [7]. Mastitis and breast abscess have potential negative impact on infant feeding. Effective managements such as education, counselling and monitoring are essential to control the discomfort and decrease the likelihood of discontinuation of breastfeeding [8].

Lactational mastitis is an acute inflammatory process of affected mammary duct and peripheral connective tissue by pathogenic bacteria, which is clinically characterized by a red, hot, swollen and wedge-shaped area of the breast with influenza-like symptoms, such as fever and malaise [5]. It can lead to stop breast-feeding and use alternative formula to feed infants, which reduce the protective effect of breastfeeding on mother and baby. Based on this, prevention of lactational acute mastitis to ensure breastfeeding rate and extend the protection of breastfeeding is extremely necessary. According to substantial epidemiologic evidence [9], lactational mastitis is a complex disease caused by sociodemographic, biophysical and psycho-social factors [10]. More and more literatures reveal that maternal age, education, residence, physical status, breastfeeding behaviors, psychological mood and infant oral development defects have been widely considered as potential risk factors for the lactational mastitis [11].

In summary, lactational mastitis is the focus of renewed attention with high economic, social and public health impact. The aim of this research is to investigate and clarify the risk factors associated with the occurrence of breast mastitis in Chinese women, and to establish risk assessment model to identify and warn the individuals susceptible to lactational mastitis early. Furthermore, our aim is also to standardize propaganda and education and to formulate preventive measures, which will allow practitioners to provide appropriate management advice, scientific treatment strategies and effectively individual care.

## Materials and methods

### Participants’ recruitment

The patients who participated in this case–control study were recruited at breast surgery department of Shandong Cancer Hospital and Qilu Hospital of Shandong University between June 2016 and December 2017. The diagnosis was made according to the Academy of Breastfeeding Medicine (ABM) diagnostic criteria for lactational mastitis [6,12], which were defined as self-reported symptoms of tender, hot, swollen, wedge-shaped area of breast, accompanied by one or more of the following [13]: (1) an elevated temperature 38.5°C or greater, (2) one of the constitutional symptoms of fever (body aches, headaches and chills), (3) total number of white blood cells and neutrophils increased, WBC > 10.0 × 10^9^/l and NEUT > 7.0 × 10^9^/l, (4) axillary lymph node enlargement. Symptoms must be present for a minimum duration of 24 h. Controls enrolled in the present study were female who attended post-partum follow up clinics at Health Management Center of Qilu hospital and must meet the following criteria: (1) age ranging from 20 to 40, (2) breastfeeding experience without acute mastitis during lactation, (3) stopped breastfeeding within 2 years.

### Estimate of sample size needed

The sample size of the case–control study was estimated with PASS 11 software. According to previous literature, the average proportion of multiple exposures in control group was approximately 20% [14,15]. Meanwhile, we formulated the expected odds ratio (OR) = 2.0, the test level α = 0.05, β = 0.10, and the test performance 1 - β = 0.90. Based on the assumption above, the sample size of the case group and the control group was calculated.

### Questionnaire

Based on the previous studies and a review of the literatures [15,16], a questionnaire was developed to collect data on general sociodemographic, psychosocial and puerperium characteristics. The questions included maternal age, residence, occupation, education, birth and breastfeeding history, family history, alcohol or smoking status and delivery mode. Psychosocial variables referred to the maternal mood, such as pleasure, happiness, depression and irritability. Puerperium characteristics concerned the time of first breastfeeding, heteroplasia of nipple, frequency of nipple cleaning, sleep posture, breast trauma, maternal physical health, antibiotics use, infant feeding and sucking patterns, tongue-tie and oral thrush. Moreover, three types of nipple's dysplasia including nipple retraction, nipple applanation and large nipple are defined. The normal nipple is 0.8–1.5cm in diameter and 1.5–2 cm in height. Nipple retraction means that the nipple is sunken in the areola and does not protrude from the outside. Nipple applanation refers to the height of the nipple <0.5 cm. Large nipple refers to the diameter of the nipple >2.5 cm. Poor connection between mother and baby in the questionnaire is mainly due to improper embrace position, nasal obstruction and other causes of infants that cannot correctly include nipple.

In addition to considering the logic, the items listed in the questionnaire also should be arranged in order and preset as three dimensions of delivery and postpartum characteristics, breastfeeding behaviors and characteristics, infants practices and characteristics. The reliability and validity of the scale were measured by Cronbach’s α coefficient and exploratory factor analysis respectively. As far as possible, we selected simple and understandable questions for respondents to simplify the contents and ensure the accuracy of the results. Furthermore, minimized personal information related topics, and reduced the incidence of such cases that were loss of compliance due to fear of privacy leakage, so as to ensure the authenticity of the questionnaire.

### Design process and quality control

**Figure 1:**
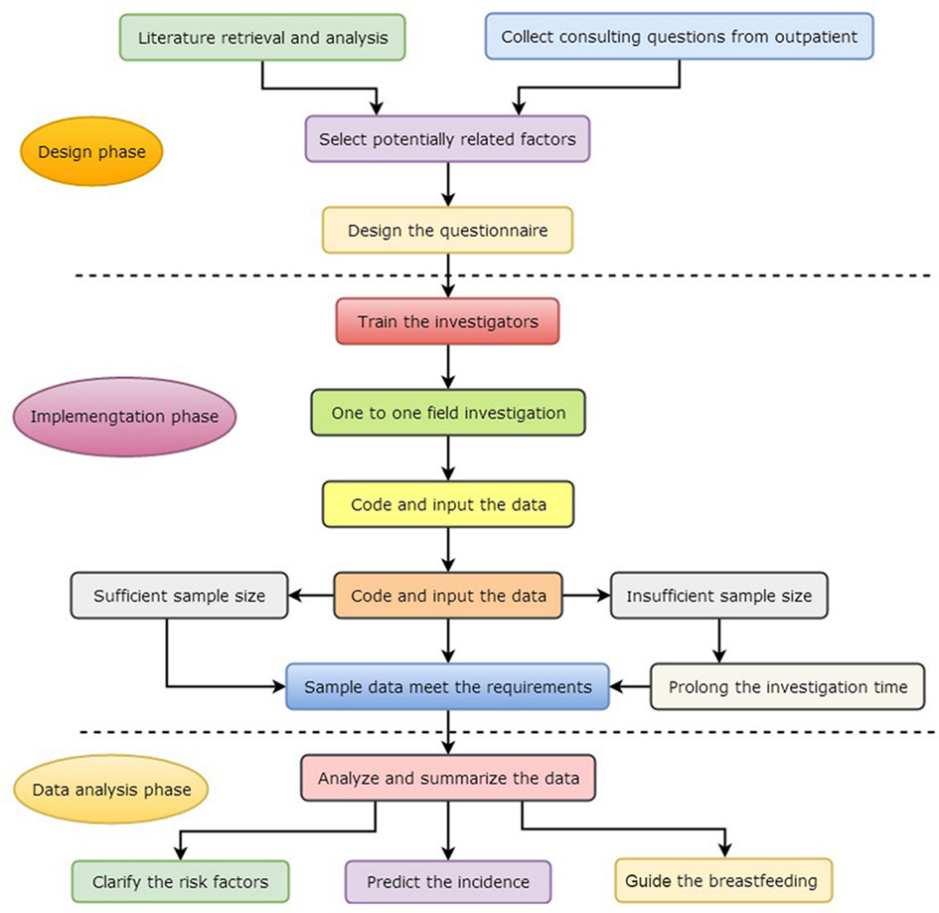
Implementation flow chart in the research The procedures of research were divided into design phase, implementation phase and data analysis phase. Each variable in the questionnaire was assigned, and the original data were encoded and entered into the Excel table, which were imported into the SPSS software package for statistical analyses. Strict quality control was essential to avoid measurement bias from investigators and respondents.

The procedures of research were divided into design phase, implementation phase and data analysis phase (Figure 1). (1) Design phase: on the basis literatures, we designed the research program, formulated research content and work plan, formed a unified and standardized questionnaire survey, which were revised by breast specialists and printed uniformly in the end. (2) Implementation phase: one to one surveys were conducted on the subjects who meet the inclusion criteria. Subsequently, checked the original data, stored and collated each completed questionnaire. (3) Data analysis phase: each variable in the questionnaire was assigned, and the original data were encoded and entered into the Excel table, which were imported into the SPSS software package for statistical analyses.

Strict quality control was essential to avoid measurement bias from investigators and respondents. Uniform training was provided to all investigators on the survey requirements, the definition of the contents and the methods of the investigation. In the field study, direct inquire after was adopted to make the inquiry method and time the same for each participant. Both parties established good communication to ensure that the questions in the questionnaire were fully understand by the subjects, so as to obtain objective and accurate data.

### Ethical considerations and informed consent

The ethical approval of the survey was obtained from the Human Ethics Committee of Shandong Cancer Hospital. Every subject signed a written consent. The original questionnaires and data were involved special person in charge, special closet for storage, special computer for registration and encryption.

### Statistical analysis

Participants’ baseline characteristics according to mastitis status were presented using proportions for categorical variables. Comparisons of categorical variables in both mastitis and control groups were done using chi-square test (χ2). To analyze the association between mastitis and the potential risk factors, a multivariate logistic regression model was constructed and odds ratio (OR) with 95% confidence interval (CI) were calculated. To assess abilities of combined variables with significant differences in multivariate logistic regression to predict incidence of mastitis, ROC curves, which correlated true- and false-positive rates (sensitivity and 1-specificity), were constructed. In addition, ROC curves established by risk factors from American ABM guidelines for treatment of mastitis was used as reference. Both AUCs (area under the curve) were calculated and the statistical significance of differences between the two AUCs also was determined. All statistical analyses were performed with SAS 9.3 and a two-sided *P* < 0.05 was considered as statistically significance.

## Results

### Calculation and collection of samples

**Figure 2:**
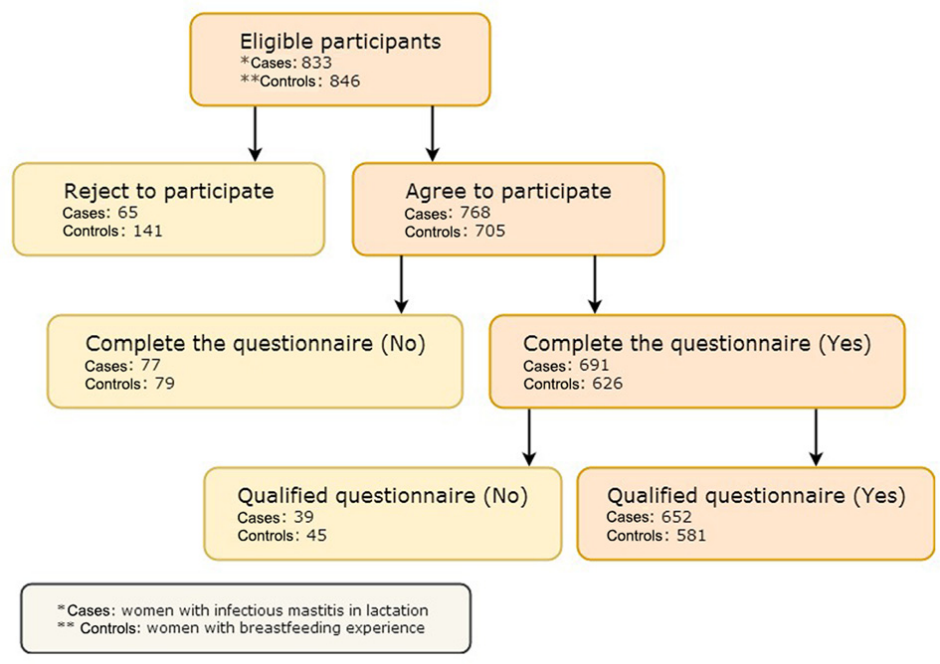
The flow chart of 1679 respondents distribution In the case, 768 patients agreed to participate and only 691 patients completed the questionnaires, the other 77 cases were unfinished due to poor communication and cooperation during the investigation and no response/lost follow-up. Finally, 652 cases were qualified, the remaining 39 cases were excluded owing to incomplete information collection/multiple selection or mission of the options in the questionnaire. In the control, 846 healthy women with breastfeeding experience were recruited into our study as controls and 581 females completed the investigation in the end. There were finally 1233 patients recruited as cases or controls, and 466 respondents left during study period due to a variety of reasons. *Cases: women with infectious mastitis in lactation. **Controls: women with breastfeeding experience

The sample size of case group and control group calculated by PASS 11 software is 230. Taking into account the 10% rate of lost follow-up and the 10% rate of unqualified questionnaires, at least 289 samples are required for each group. Based on the steps and procedures mentioned in the method, we approached 833 eligible patients who had acute lactational mastitis. Among them, 768 patients agreed to participate and only 691 patients completed the questionnaires, the other 77 cases were unfinished due to poor communication and cooperation during the investigation and no response/lost follow-up. Finally, 652 cases were qualified, the remaining 39 cases were excluded owing to incomplete information collection/multiple selection or mission of the options in the questionnaire. Similar to the above processes, 846 healthy women with breastfeeding experience were recruited into our study as controls and 581 females completed the investigation in the end (Figure 2). There were finally 1233 patients recruited as cases or controls, and 466 respondents left during study period due to a variety of reasons.

### Reliability and validity test of questionnaire

Before the questionnaire was officially issued, we invited 32 patients with mastitis and normal women with previous breastfeeding experience to conduct a small-scale test. The researchers adjusted the content and structure of the questionnaire according to the feedback and calculated the reliability and validity of the questionnaire.

The reliability of the scale measured by Cronbach’s α coefficient was 0.932. By the score with expert review, the content validity index of the scale was 0.897, and with the Bartlett test, the χ2 = 6684.148, *P* < 0.001, and Kaiser–Meyer–Olkin (KMO) = 0.858, that represented good structural validity.

### General characteristics of research objects

The study enrolled 652 patients with lactational mastitis and 581 healthy women after completing all the questionnaires designed to collect retrospective information about different factors related to mastitis. In descriptive analyses, the average age was 29.89 ± 3.37 for cases and 30.26 ± 3.78 for controls. Many essential relations were also established between the age range at last delivery, residence, occupation, education, delivery number and mode, mastitis in previous breastfeeding, maternal body mass index (BMI), smoking, drinking and acute infectious mastitis.

Concerning sociodemographic characteristics, women in the case group were more likely to have Bachelor or below (*P* = 0.010), primiparity (*P* = 0.018), caesarean section (*P* = 0.011), history of mastitis (*P* < 0.001) comparing with the control. Meanwhile, there were no significant differences between case and control subjects with regard to age at last delivery (*P* = 0.318), residence (*P* = 0.087), occupation (*P* = 0.163), maternal BMI (*P* = 0.272), smoking (*P* = 0.076) and drinking (*P* = 0.889). Information about the main sociodemographic characteristics of the samples is shown in Table 1.

**Table 1:** Sociodemographic characteristics of 1233 women who participated in the case–control study

Variables	Case, *n* (%)	Control, *n* (%)	Crude OR	95% CI	*P* value
Age range at last delivery					0.318
<25	37 (5.7)	23 (4.0)	0.791	0.396–1.580	
25–29	411 (63.0)	361 (62.1)	1.118	0.694–1.802	
30–35	162 (24.8)	164 (28.2)	1.288	0.778–2.135	
>35	42 (6.4)	33 (5.7)	Reference		
Residence					0.087
Urban	487 (74.7)	458 (78.8)	0.713	0.440–1.476	
Rural	165 (25.3)	123 (21.2)	Reference		
Occupation					0.163
Professional women	416 (63.8)	388 (66.8)	0.862	0.559–1.143	
Housewife	236 (36.2)	193 (33.2)	Reference		
Education					0.010*
Bachelor or below	588 (90.2)	505 (86.9)	1.248	1.055–1.475	
Master or above	64 (9.8)	76 (13.1)	Reference		
Total	652	581			

* Significant at α ≤ 0.05

### Univariate analysis and multivariate logistic regression analyses

Factors significantly associated with mastitis risk in a previous univariate analysis (Table 2) were included in the logistic regression and shown in Table 3. In terms of past medical history, there was no education-related significant difference, but mastitis in previous breastfeeding was obviously shown by women with mastitis compared to controls (OR = 4.945, 95%CI = 3.123–7.829). Between the two groups, primiparous females (OR = 2.233, 95%CI = 1.602–3.113) and vaginal delivery (OR = 0.702, 95%CI = 0.527–0.934) were respectively observed to be barely significant risk factor and protective factor. Sleeping posture also revealed significant difference comparing cases with controls. Lateral position was found remarkably more frequent in the mastitis group than in the control group (OR = 1.502, 95%CI = 1.133–1.991).

**Table 2:** Univariate analyses of 1233 women who participated in the case–control study

Variables	Cases, *n* (%)	Controls, *n* (%)	Crude OR	95% CI	*P* value
Delivery and postpartum characteristics					
Delivery number					0.018*
Primiparous	405 (62.1)	322 (55.4)	2.669	1.322–4.078	
Multiparous	247 (37.9)	259 (44.6)	Reference		
Delivery mode					0.011*
Vaginal	395 (60.6)	393 (67.6)	0.758	0.599–0.959	
Caesarean section	257 (39.4)	188 (21.2)	Reference		
Mastitis in previous breastfeeding**					<0.001*
Yes	132 (53.4)	56 (21.6)	1.582	1.258–1.988	
No	115 (46.6)	203 (78.4)	Reference		
First contact with child					0.001*
≤ 1 h	577 (88.5)	547 (94.1)	0.478	0.314–0.729	
> 1 h	75 (11.5)	34 (5.9)	Reference		
Separation of mother-infant					0.013*
> 24 h	61 (9.4)	32 (5.5)	3.226	1.887–5.525	
≤ 24 h	591 (90.6)	549 (94.5)	Reference		
Smoking / Drinking					0.869
Yes	21 (3.2)	17 (2.9)	0.906	0.473–1.734	
No	631 (96.8)	564 (97.1)	Reference		
Breastfeeding behaviors and characteristics					
Feeding type					0.002*
Exclusive	463 (71.0)	461 (79.4)			
Mixed	181 (27.8)	118 (20.3)			
Formula	8 (1.2)	2 (0.3)	Reference		
Nipple’s heteroplasia					<0.001*
Nipple retraction	67 (10.3)	6 (1.0)	12.530	5.378–29.192	
Nipple applanation	122 (18.7)	52 (9.0)	4.617	1.883–11.324	
Large nipple	16 (2.5)	6 (1.0)	4.063	1.156–14.277	
No	447 (68.6)	517 (89.0)	Reference		
Cracked nipple					<0.001*
Yes	389 (59.7)	103 (17.7)	6.864	5.270–8.940	
No	263 (40.3)	478 (82.3)	Reference		
Breast trauma by external force					<0.001*
Yes	129 (19.8)	65 (11.2)	7.152	5.296–9.656	
No	523 (80.2)	516 (88.8)	Reference		
Breastfeeding behaviors and characteristics					
Sleeping posture					0.002*
Prone position	28 (4.3)	12 (2.1)	3.630	1.748–7.602	
Lateral position	304 (46.6)	233 (40.1)	2.650	1.546–3.867	
Supine position	320 (49.1)	336 (57.8)	Reference		
Clean nipple before breastfeeding					0.002*
Yes	457 (70.1)	452 (77.8)	0.449	0.324–0.624	
No	195 (29.9)	129 (22.2)	Reference		
Breast pump					0.007*
Yes	442 (67.8)	351 (60.4)	5.940	4.638–7.608	
No	210 (32.2)	230 (39.6)	Reference		
Non-medical staff massage					0.008*
Yes	305 (46.8)	228 (39.2)	7.115	5.506–9.194	
No	347 (53.2)	353 (60.8)	Reference		
Maternal BMI					0.272
< 18.5	35 (5.4)	19 (3.3)	1.779	0.961–3.292	
18.5–22.9	117 (17.9)	113 (19.4)	1.569	0.866–2.842	
23.0–25.0	202 (31.0)	172 (29.6)	1.712	0.957–3.064	
> 25.0	298 (45.7)	277 (47.7)	Reference		
Throat infection					0.014*
Yes	116 (17.8)	74 (12.7)	1.483	1.081–2.034	
No	536 (82.2)	507 (87.3)	Reference		
Anemia					0.032*
Yes	73 (11.2)	44 (7.6)	1.541	1.041–2.281	
No	578 (88.8)	537 (92.4)	Reference		
Unauthorized oral antibiotics					0.039*
Yes	122 (18.7)	83 (14.3)	1.951	1.456–2.615	
No	530 (81.3)	498 (85.7)	Reference		
Psychological mood					0.001*
Negative	498 (76.4)	393 (67.6)	3.771	2.806–5.068	
Positive	154 (23.6)	188 (32.4)	Reference		
Infants practices and characteristics					
Sucking manners					<0.001*
Nipple sucking	146 (22.4)	72 (12.4)	3.734	2.777–5.022	
Nipple shields	32 (4.9)	9 (1.5)	1.381	0.602–3.169	
Areola sucking	474 (72.7)	500 (86.1)	Reference		
Sleep with sucking					0.001*
Yes	349 (53.5)	255 (43.9)	4.159	3.239–5.342	
No	303 (46.5)	326 (56.1)	Reference		
Connection difficulty					<0.001*
Yes	114 (17.5)	58 (10.0)	4.598	3.056–6.920	
No	538 (82.5)	523 (90.0)	Reference		
Oral dysplasia					0.008*
Yes	58 (8.9)	29 (5.0)	1.571	1.013–2.437	
No	594 (91.1)	552 (95.0)	Reference		

* Significant at α ≤ 0.05

** Data from primiparous women were excluded for this analysis.

**Table 3:** Factors significantly associated with mastitis risk in a previous univariate analysis that were included in the multivariate logistic regression

Variables	Adjusted OR	95%CI	*P* value
Education			
Bachelor or below	1.237	0.795–2.109	0.315
Master or above	Reference		
Delivery number			
Primiparous	2.233	1.602–3.113	<0.001*
Multiparous	Reference		
Delivery mode			
Vaginal	0.702	0.527–0.934	0.015*
Caesarean section	Reference		
Sleeping posture			
Prone position	2.105	0.942–4.703	0.007*
Lateral position	1.502	1.133–1.991	
Supine position	Reference		
Mastitis in previous breastfeeding			
Yes	4.945	3.123–7.829	<0.001*
No	Reference		
Nipple's heteroplasia			
Nipple retraction	9.114	3.629–22.884	
Nipple applanation	1.632	1.070–2.490	<0.001*
Large nipple	1.853	0.588–5.845	
No	Reference		
Cracked nipple			
Yes	5.807	4.334–7.782	<0.001*
No	Reference		
Clean nipple before breastfeeding			
Yes	0.681	0.499–0.929	0.015*
No	Reference		
Breast pump			
Yes	1.348	1.015–1.790	0.039*
No	Reference		
Non-medical staff massage			
Yes	1.286	0.975–1.695	0.074
No	Reference		
Breast trauma by external force			
Yes	1.845	1.256–2.711	0.002*
No	Reference		
Sucking manners			
Nipple sucking	1.664	1.141–2.427	0.007*
Nipple shields	2.486	0.977–6.327	
Areola sucking	Reference		
Sleep with sucking			
Yes	1.460	1.110–1.921	0.007*
No	Reference		
Connection difficulty			
Yes	1.015	0.662–1.555	0.946
No	Reference		
Oral dysplasia			
Yes	2.026	1.160–3.539	0.013*
No	Reference		
Anemia			
Yes	1.378	0.846–2.245	0.198
No	Reference		
Throat infection			
Yes	1.552	0.961–2.507	0.072
No	Reference		
Unauthorized oral antibiotics			
Yes	1.057	0.666–1.676	0.814
No	Reference		
Psychological mood			
Negative	1.328	0.981–1.797	0.066
Positive	Reference		
First contact with child			
≤ 1h	0.565	0.340–0.938	0.027*
> 1h	Reference		
Separation of mother-infant			
> 24h	1.613	0.933–2.790	0.087
≤ 24h	Reference		

* Significant at α ≤ 0.05

Women in the mastitis group were more likely to have nipple’s dysplasia, such as nipple retraction (OR = 9.114, 95%CI = 3.629–22.884) and applanation nipple (OR = 1.632, 95%CI = 1.070–2.490). The more important concern is that cracked nipple (OR = 5.807, 95%CI = 4.334–7.782) would prominently lead to acute mastit is in lactating women. In contrast, cleaning nipple before breastfeeding regularly (OR = 0.681, 95%CI = 0.499–0.929) may effectively avoid the occurrence of infectious mastitis. Considering the breastfeeding behaviors and practices, use of breast pump (OR = 1.348, 95%CI = 1.015–1.790) and breast trauma by external force (OR = 1.845, 95%CI = 1.256–2.711) were mastitis risk factors. Nevertheless, there was no statistically difference between cases and controls related to the non-medical staff massage.

At the same time, some associations deserving attention are variables of infants practices and characteristics. For instance, sucking manners (OR = 1.664, 95%CI = 1.141–2.427) and sleep with sucking (OR = 1.460, 95%CI = 1.110–1.921) were significantly associated with infectious mastitis. Compared with the way of areola sucking, nipple sucking (OR = 1.664, 95%CI = 1.141–2.427) were more administrated to children in the case group than in the control group. A higher risk of mastitis was noted when the infant suffered from tongue-tie (OR = 2.026, 95%CI = 1.160–3.539). In addition, connecting difficulty between mother and baby did not yield a significant difference in the case–control study.

As to the maternal physical condition and psychological mood, the factors of anemia and throat infection were illustrated without significant variation. Similarly, uses of unauthorized oral antibiotics and negative emotion were not associated with mastitis that was opposite to the views reported in other literatures [16,17]. Contact with infant after birth within 1 h accounted for a greater proportion in control group and protect women from mastitis (OR = 0.565, 95%CI = 0.340-0.938). Nevertheless, separation from mother longer than 24 h had no obvious statistics difference in both case and control groups.

### Evaluation discriminant model with ROC

**Figure 3:**
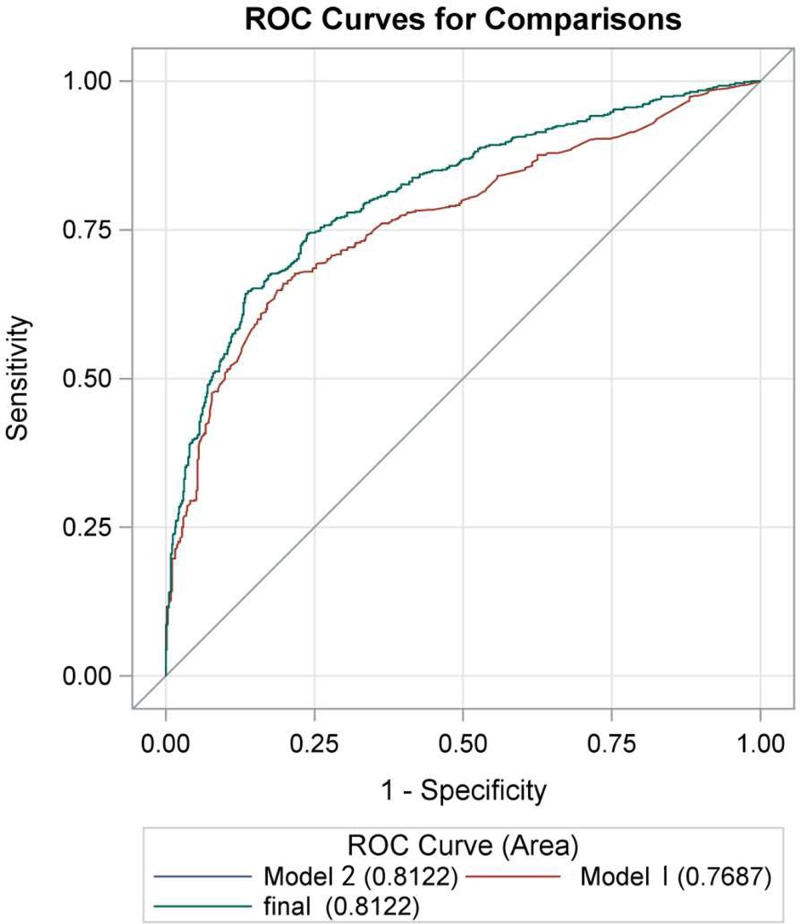
Receiver operating characteristic (ROC) curve for logistic regression model The model 1 was established according to the related factors including mastitis in previous breastfeeding, abnormal nipple’s development, cracked nipple, breast trauma, poor connection of mother-infant, unauthorized oral antibiotics, tongue-tie, maternal BMI, psychologic status, and education, of which AUC was 0.7687, and 95%CI was 0.7424–0.7950. The model 2 was set up based on the following factors such as deliver mode, deliver number, sleeping posture, breast pumps, clean nipple before breastfeeding, first contact with child within 1 h, sucking manners, sleep with sucking, non-medical staff massage and tongue-tie, and combined with the indicators from model 1 to perform ROC curve analysis, of which AUC was 0.8122, and 95%CI was 0.7885–0.8360. Models 1 and 2 are statistically significant for the ability to screen and discriminate mastitis (χ^2^ = 33.2405, *P* < 0.0001).

According to the ABM treatment guidelines of mastitis [6] and common related factors, including mastitis in previous breastfeeding, nipple’s heteroplasia, cracked nipple, breast trauma by external force, poor connection of mother–infant, unauthorized oral antibiotics, tongue-tie, maternal BMI, psychologic status, and education, we have established discriminant model 1 using ROC curve, of which AUC was 0.7687, and 95%CI was 0.7424–0.7950. Additionally, screened variables with logistic regression analysis, for instance, deliver mode, deliver number, sleeping posture, breast pumps, clean nipple before breastfeeding, first contact with child within 1 h, sucking manners, sleep with sucking, non-medical staff massage and tongue-tie, combined with the indicators from model 1 to perform ROC curve analysis and set up model 2, which displayed the area under the ROC curve was 0.8122 (95%CI = 0.7885–0.8360). Models 1 and 2 are statistically significant for the ability to screen and discriminate mastitis (χ^2^ = 33.2405, *P* < 0.0001), which stated that the model 2 presented a good fit and a terrific adjustment (Figure 3).

## Discussion

According to epidemiologic studies, breastfeeding has been proven to have more short- and long-term benefits, which is highly associated with reduced risk of acute otitis media, nonspecific gastroenteritis, severe lower respiratory tract infections, childhood leukemia, and sudden infant death syndrome for babies [18,19] and Type 2 diabetes, breast and ovarian cancers for mothers [18,20]. In spite of proven profits and repeated emphasis, only 42% women begin breastfeeding within an hour of birth [21], and the rate of exclusive breastfeeding (EBF) among children less than 6 months of age is only 36% globally [22,23]. WHO Member States have agreed six Global Targets, of which one target is to increase the rate of EBF in the first 6 months to at least 50% by 2025 [24].

Over the years, acute infectious mastitis has still been considered to be the main cause of temporary and permanent breastfeeding weaning in lactation. Comprehensive and systematic risk factors analyses and predictive evaluations of acute mastitis have been less reported. There is consensus in the literatures that the most common factors for mastitis depend on a large number of determinants included maternal education and employment, traditional healthcare practices, breastfeeding behaviors and characteristics [11,15,16,25]. The importance of various factors to the initiation of mastitis can be different due to socioeconomic status and cultural background [24].

The purpose of the case–control investigation is to identify the potential risk and protective factors related to mastitis. Among them, the first contact between mother-infant within 1 h could reduce the risk of mastitis (OR = 0.565, 95%CI = 0.340–0.938). That is to say, if separation from the maternity and newborn after birth prolonged due to hypoxia, hospitalization or any other reasons may increase the risk of mastitis in lactation. The initial 1–2 h after birth is the best time for sucking action and foraging reflex [26]. It is also a sensitive period to set up effective breastfeeding [27]. Early exposure and sucking can continuously stimulate the secretion of prolactin (PRL) in the anterior pituitary, which could establish breastfeeding as early as possible. For this reason, we should highlight the crucial importance of early skin-to-skin contact of mother and baby [28] to set up a powerful sucking ability, which can effectively empty milk in time and avoid mastitis.

This finding is consistent with previous evidence [29,30] that the primiparous women were found significantly more frequent in the mastitis group than in the control group (OR = 2.233, 95%CI = 1.602–3.113). Primipara without previous experience of childbirth lacks healthcare knowledge and preventive measures for postpartum rehabilitation, breastfeeding and lactation mastitis, but also faces the adaptation process of new roles [31]. Therefore, the primipara is in urgent need of comprehensive and meticulous guidance [32] by strengthening interactive education about the pathogenesis and prevention of lactational mastitis, so that when suffering from nipple dysplasia, cracked nipple, engorgement, plugged ducts and galactostasis, they could take effective actions to alleviate the uncomfortable symptoms of maternal breast distention and pain. The study from Holmes demonstrates that a formal breastfeeding curriculum can improve physician’s knowledge and attitudes, change practice around breastfeeding care, also reduce the risk of mastitis and improve clinical outcomes [33].

For the last 20 years, a few research have reported the relationship between the delivery mode and acute lactational mastitis [11,15,16]. From our study, it can be seen that the vaginal delivery is a protective factor (OR = 0.702, 95%CI = 0.527–0.934) for acute lactational mastitis, and the cesarean section is a risk factor in contrast. The increased morbidity of mastitis due to the mode of childbirth usually occurs during puerperium (6 weeks after childbirth). The possible reasons are as follows. First, the puerpera with cesarean section are afraid of feeding the newborn because of many discomfort factors such as postpartum pain [34], infusion, indwelling catheter and compulsive position, which lead to poor discharge of milk and galactostasis. Second, the women who had undergone cesarean section may result in low immunity and flora imbalance. With synergistic effect of opportunistic pathogens, the proliferation of pathogenic bacteria has happened in the damaged nipple or blocked duct, which accelerates the progress of mastitis. Moreover, the development of new strategies for mastitis management based on probiotics is particularly appealing [35].

Significantly, a previous history of lactational mastitis is also a strong mastitis predictor in our study, and this factor is associated with a nearly 5-fold risk of mastitis in the multivariate analysis (OR = 4.945, 95%CI = 3.123–7.829). In the past, scholars used to think that lactation mastitis was a simple infectious disease, and now this view is facing a challenge. Its pathogenesis has changed from a simple infectious disease to inflammatory reaction [9]. The susceptibility and severity of mastitis are not related to the number of infectious pathogenic bacteria, but related to breast morphology, mammary duct development, the immune response of the body [36], and these factors can lead to mastitis in women time and again.

Nipple is located in the posterior part of the baby’s oral cavity while sucking, which can effectively stimulate the sensory nerve on the nipple, thus promoting lactation reflex and avoiding nipple injury. As a result, the WHO, the UNICEF and Maternal and Child health Department of National Health Commission of the People’s Republic of China have successively promulgated the breastfeeding handbook, which promote and guide breast feeding with nipple and areola, so as to better discharge milk and avoid nipple pain and breakage [37]. To conclude, our findings have supported that nipple sucking (OR = 1.664, 95%CI = 1.141–2.427), nipple heteroplasia such as nipple retraction (OR = 9.114, 95%CI = 3.629–22.884) and nipple applanation (OR = 1.632, 95%CI = 1.070–2.490) were significantly more widely administered to women reporting acute mastitis. In theory, the skin of nipple retraction is delicate and the stratum corneum is not thick owing to less external stimulation at ordinary times. As a result, tender nipples are prone to chapping after sucking, where is invaded by the pathogen to the mammary lobules and their fat and fibrous tissue, and eventually cause the acute mastitis. There was a general consensus with previous studies [16] that tongue-tie (OR = 2.026, 95%CI = 1.160–3.539) strongly links to lactational mastitis. In conclusion, mothers need to be fully aware of and correct their nipple before giving birth, make preventive measures earlier, and better learn nipple care knowledge to compensate for potential mastitis risk caused by defects in congenital dysplasia of nipples.

In the present study, the demonstrated improvement was likely a result of correlation between breast pump (OR = 1.348, 95%CI = 1.015–1.790), breast trauma by external force (OR = 1.845, 95%CI = 1.256–2.711), sleeping lateral position (OR = 1.502, 95%CI = 1.133–1.991) and infectious mastitis. Because mammary gland ducts are filled with milk, the tube wall is thin, and the subcutaneous tissue of the mammary gland is rich in lymphatic vessels and capillaries, it is prone to cause local breast tissue damage and edema in the effect of external force compression, collision, inappropriate massage and infant kicking. When sleeping on the side, the milk remains in the breast tube near the side of bed due to gravity, and the breast is compressed leading to galactostasis. Meanwhile, the frequent and improper use of breast pumps was a relevant mastitis risk factor [38], which may give rise to tissue damage, ache, and nipple trauma and if not sterilized on time they could be a source of pathogenic bacteria.

There are intimate links between bacterial invasion with occurrence and development of acute mastitis during lactation. First of all, the bacteria on the skin surface can intrude through the cracked nipple and areola, and retrograde to interlobular mammary gland. Second, the microorganisms in the baby’s mouth enter the mammary duct and stay in it [39]. Finally, some studies suggest [16,40] that there is an endogenous pathway existing in the source of microbes of milk, which involve in bacterial infections in other parts of the postpartum (such as respiratory infections, acute tonsillitis etc.) through lymphatic system and blood system to the breast. The results of the present study demonstrate that long suck by babies while they sleep was significantly more widely administered to women reporting lactational mastitis (OR = 1.460, 95%CI = 1.110–1.921), while cleaning nipples before or after breastfeeding can reduce bacterial invasion and the incidence of mastitis (OR = 0.681, 95%CI = 0.499–0.929).

Previous studies [41] have confirmed that negative emotions can activate hypothalamus–pituitary–adrenal (HPA) axis and sympathetic nervous system (SNS) axis can up-regulate the levels of glucocorticoid (GCs) and catecholamines (CAs) respectively, causing raised blood pressure and vasoconstriction, which reduce the blood supply for the breast, and the secretion of milk. In addition to this, pessimism can also affect the release of prolactin, which may diminish the possibility of milk deposition. But at the same time, another research [42] displayed that negative emotions can lead to a decrease in secretory immunoglobulin A(SIgA) levels, resulting in weakening the body resistance to bacteria. The pathogenic factors of acute mastitis in lactation are milk siltation and bacterial infection. Although negative emotions decreased the former, it also enhanced the role of the latter. At present, it is still unclear which of these two factors play the dominant role. Our result revealed that no significance was found between the patients and the controls with regard to psychological mood. In any case, the mother should remain happy and optimistic during pregnancy and lactation, which is essential for strengthening immunity, reducing illness and raising babies [43,44]. As well as the family, hospital and society also need to give mothers thoughtful care and guidance.

Although unauthorized oral antibiotics was not an independent risk factor for mastitis in this analysis. Theoretically, use of large amount of antibiotics without indication can reduce maternal resistance and lead to microbial imbalance of breast bacteria, and the potential pathogens have overgrown as losing the inhibition of other bacteria [34]. Antibiotics are often used in the treatment of mastitis, but have not been popular or proven effective as a preventative agent. Increasing research indicates that specific probiotic bacteria possess significant anti-inflammatory properties and supports their potential use as immunomodulatory agents [45]. Supplementation with lactobacilli and probiotics may eliminate the risk of mastitis in women and prevent breast infection. In future, we need to pay more attention to the management of antibiotic resistant bacteria, select lactobacilli strains isolated from breast milk, evaluate the effectiveness of oral probiotics for the prevention and treatment of mastitis in breastfeeding women.

The results of our study demonstrated that relevant factors related to an increased risk of infectious mastitis in Chinese breastfeeding women, which will allow practitioners to provide appropriate management advice and professional health care. It is important to investigate preventive measures in order to maintain and increase breastfeeding exclusivity and duration, especially for high-risk individuals. There are still many questions to answer about lactational mastitis, but work is in progress to broaden our knowledge in this relevant Public Health issue.

## Data Availability

The datasets used and/or analyzed during the current study are available from the corresponding author on reasonable request.

## Abbreviations

ABM: academy of breastfeeding medicine
BMI: body mass index
CI: confidence interval
EBF: exclusive breastfeeding
OR: odds ratio
ROC: receiver operating characteristic
UNICEF: united nations international children's emergency fund
WHO: world health organization
